# Immune Profile of SARS-CoV-2 Variants of Concern

**DOI:** 10.3389/fdgth.2021.704411

**Published:** 2021-07-09

**Authors:** Caterina A. M. La Porta, Stefano Zapperi

**Affiliations:** ^1^Center for Complexity and Biosystems, University of Milan, Milan, Italy; ^2^Department of Environmental Science and Policy, University of Milan, Milan, Italy; ^3^CNR - Consiglio Nazionale delle Ricerche, Istituto di Biofisica, Genoa, Italy; ^4^Department of Physics, University of Milan, Milan, Italy; ^5^CNR - Consiglio Nazionale delle Ricerche, Istituto di Chimica della Materia Condensata e di Tecnologie per l'Energia, Milan, Italy

**Keywords:** SARS-CoV-2, T cells, MHC, polymorphism, virus mutation

## Abstract

The spread of the current Sars-Cov-2 pandemics leads to the development of mutations that are constantly monitored because they could affect the efficacy of vaccines. Three recently identified mutated strains, known as variants of concern, are rapidly spreading worldwide. Here, we study possible effects of these mutations on the immune response to Sars-Cov-2 infection using NetTepi a computational method based on artificial neural networks that considers binding and stability of peptides obtained by proteasome degradation for widely represented HLA class I alleles present in human populations as well as the T-cell propensity of viral peptides that measures their immune response. Our results show variations in the number of potential highly ranked peptides ranging between 0 and 20% depending on the specific HLA allele. The results can be useful to design more specific vaccines.

## 1. Introduction

The current COVID-19 pandemic is caused by the coronavirus SARS-CoV-2, one out of seven coronaviruses known to infect humans. Not all coronaviruses cause diseases of the same severity: SARS-CoV, MERS-CoV, and SARS-CoV-2 cause serious symptoms while HCoV-HKU1, HCoV-NL63, HCoV-OC43, and HCoV-229E only produce mild symptoms (1). In order to successfully infect the host, coronaviruses must overcome the innate and the adaptive immune system (2). The individual genetic susceptibility to viral infection is known to be affected by the Human Leukocyte Antigen (HLA) system or the Major Histocompatibility Complex (MHC), a very polymorphic region of the human genome (3). For example, H1N1 flu infection was shown to be correlated with several HLAs (4, 5) and HIV infection was more pronounced in individuals with HLA-A^*^29, HLA-B^*^35, and HLA-B^*^57 (6–11). Most importantly, an association between disease severity and HLA was also revealed for patients infected by SARS-CoV (12–16).

Because experimental characterization of neoantigens is costly and time-consuming, a growing effort has been devoted to developing computational methods that could estimate the binding of individual peptides to the MHC and predict the subsequent immune response. The class I regions are located on the most telomeric part of the human MHC and include 3 highly polymorphic HLA genes, known as classical (Class Ia: HLA-A, HLA-B, and HLA-C) and 3 lowly polymorphic HLA genes, known as non-classical (class Ib: HLA-E, HLA-F, and HLA-G) (17). After viral infection, viral peptides are produced in the cytosol from proteasome activity, bind to the HLA class I molecules and are then exposed to the cellular membrane. The immune response is triggered when CD8+ T cells recognize these peptide-HLA pairs (18, 19). In a recent paper (20), we identified a set of haplotypes that bind weakly and strongly to SARS-CoV-2 peptides and assessed their prevalence in specific human subpopulations (20).

The dissemination of the SARS-CoV-2 virus in the past few months, lead to the development of many genomic variants. The two major classifications have been produced by GISAID (https://www.gisaid.org/references/statements-clarifications/clade-and-lineage-nomenclature-aids-in-genomic-epidemiology-of-active-hcov-19-viruses/) and Nextstrain (https://nextstrain.org/ncov). Nextstrain, in particular, assigns nomenclature through the designation of SARS-CoV-2 clades to label well-defined clades that reached geographic spread with significant frequency (21). According to the GISAID classification, the virus that was first detected in Wuhan in December 2019 belongs to the L clade. The next important clade is the so-called S clade appearing at the beginning of 2020. From mid-January 2020 two new variants, known as the V and G variants, appeared and rapidly became prevalent across the world.

From early December 2020 a new viral lineage, known as B.1.1.7, appeared in the UK and spread extremely rapidly, due to its increased transmissibility and longer lasting infections (22). At about the same time, the second variant of SARS-CoV-2 known as 501Y.V2 (B.1.351 lineage) appeared in South Africa. The B.1.351 variant was reported by the WHO to possess increased transmission ability and higher viral load, although it is not clear if it is associated with more severe disease (https://www.who.int/csr/don/31-december-2020-sars-cov2-variants/en/). A third variant that is spreading across the world is the lineage P.1, also known as 20J/501Y.V3, Variant of Concern 202101/02 (VOC-202101/02) or colloquially known as the Brazilian variant. The P.1 variant has 17 unique amino acid changes, ten of which are located in the spike protein. Collectively, these three variants (B.1.1.7, B.1.351, and P.1) are known as variants of concern.

Here, we use supervised neural network machine learning approaches (23) to compute binding affinities, stability and T cell propensity for peptides derived by proteosome degradation (24) from the three variants of concern of SARS-CoV-2 and 13 common HLA alleles. Similar calculations are commonly performed to identify peptides for vaccine development (25). Our results allow studying the variations in potential T-cell epitopes due to the variants of concern.

## 2. Materials and Methods

### Data and Code Availability

The source code used to obtain the results in this paper are available at https://github.com/ComplexityBiosystems/hla-covid.

### Protein Sequences

We downloaded the fasta sequence for SARS-CoV-2 (GenBank: MN908947.3). We obtained the mutated sequences by modifying the reference sequence according to the three variants of interest B.1.1.7, B.1.351, and P1. We restrict our analysis to the most abundant structural proteins (26): S,N,E,M. The resulting fasta sequences are reported as Supplementary Data.

### Identification of T Cell Epitopes

To identify potential T cell epitopes, we use NetTepi 1.0 server (https://services.healthtech.dtu.dk/service.php?NetTepi-1.0) which combines estimates for peptide-MHC binding affinity, peptide-MHC stability, and T cell propensity (23). Peptides are then ranked against a set of 200,000 natural peptides to obtain a global rank score. Here we scan all SARS-Cov-2 peptides with lengths 8–11 from the 4 structural viral proteins and retain the peptides with rank scores lower than 2%. We perform the calculations for all the available class I MHC alleles using the default values for the relative weight on stability prediction and the relative weight on T cell propensity prediction. We only consider peptides that are likely to be produced by proteasome degradation. To this end, we employ NetChop 3.1 (24) a neural network based algorithm that scans proteins for probable cleavage sites of the human proteasome.

## Results

### T Cell Propensity to SARS-CoV-2 Variants and HLA Type I Polymorphism

To investigate the variations in the T cell response to the SARS-CoV-2 variants of concern as compared with the reference virus, we use NetTepi (23), a neural network based software combining information of peptide-HLA binding, peptide-HLA stability and peptide T cell propensity. We consider the 13 HLA type I alleles available for this method, which are widely represented in human populations. In particular, the 6 HLA-A alleles are present in around 60% of the population, while the 7 HLA-B are present in around 30% of the population (20). As discussed in the Methods section, we only consider peptides that are most likely to result from proteasome degradation.

**Figure 1:**
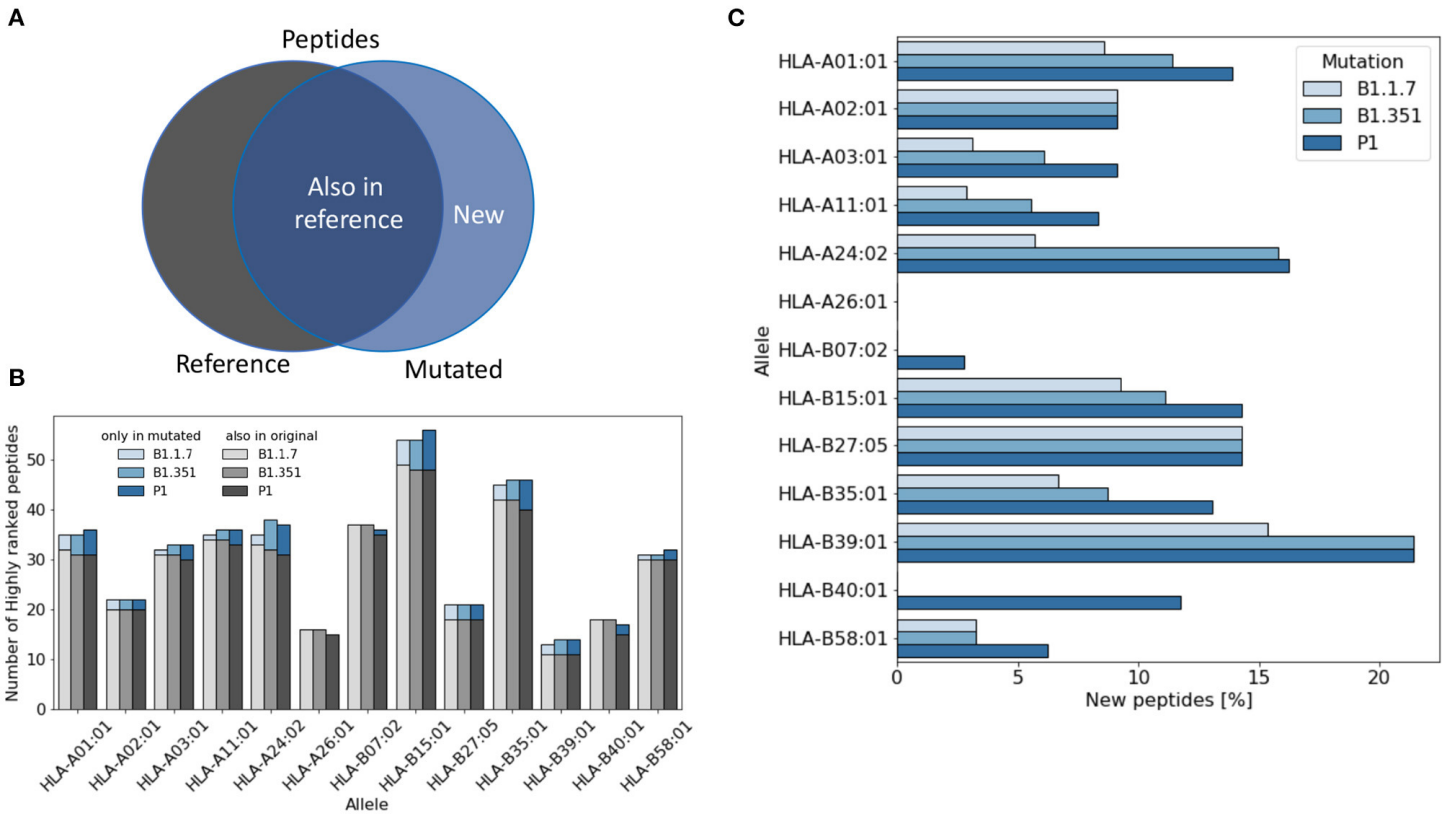
Variation of the number of T cell epitopes in virus variants. **(A)** We distinguish potential epitopes in virus variants according to their presence in the reference Sars-Cov-2 virus genome. **(B)** The total number of highly ranked peptides for each allele is reported for each virus variant and each allele. **(C)** The fraction of highly ranked peptides that were not present in the reference genome is reported for each variant and each allele.

For each virus variant, we obtain a list of highly ranked peptides that are most likely to be potential epitopes recognized by T cells. We then compare these lists with the list obtained from the reference virus and count how many potential were already present in the reference virus (Figure 1A). Figure 1B shows that the total number of potential epitopes varies only slightly for different virus variants and slightly more when comparing different HLA alleles. As illustrated in Figure 1C, the percentage of new peptides not present in the reference virus varies in the range of 0–20% depending on the HLA allele. The lowest rate of variations is found for HLA-A26 for which all the potential epitopes were already present in the reference virus, while the highest variation rate is found for HLA-B39, with more than 20% of new epitopes.

### T-Binding Affinity, Peptide Stability, and Combined Score of Highly Ranked Peptides SARS-CoV2

**Figure 2:**
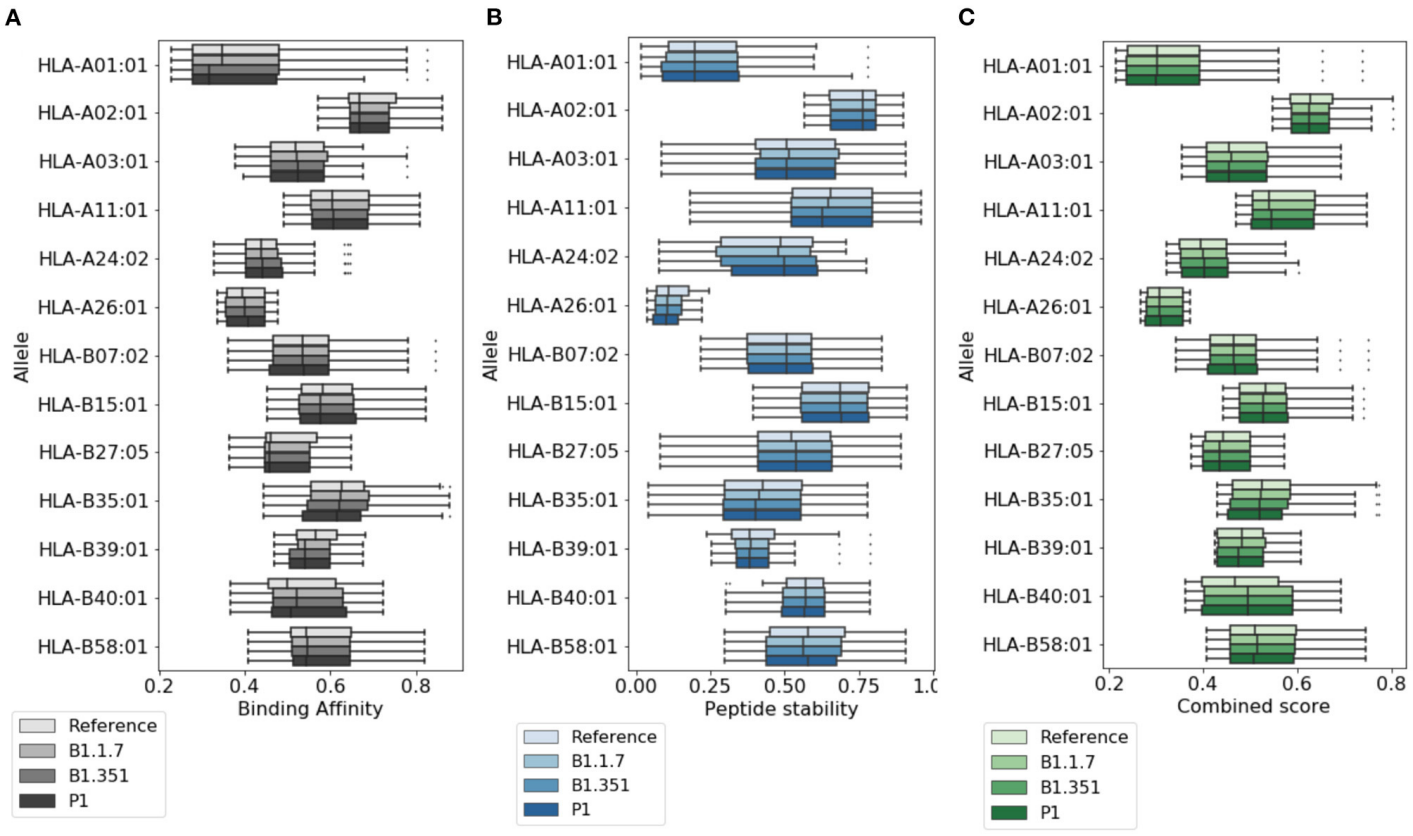
Variations of T cell epitope properties in virus variants. For the highly ranked peptides selected by NetTepi we report the boxplots for **(A)** binding affinity, **(B)** peptide stability, and **(C)** the combined score also including T cell propensity. Data are reported for different virus variants and alleles.

In Figure 2, we provide a more detailed picture of the variations in the score for the highly ranked peptides selected by NetTepi, considering binding affinity, peptide stability, and the combined score which also includes T-cell propensity. The results show that the main source of variations comes from the considered allele, while the range of values does not change significantly across the different mutations.

### Localization of Highly Ranked Peptides

**Figure 3:**
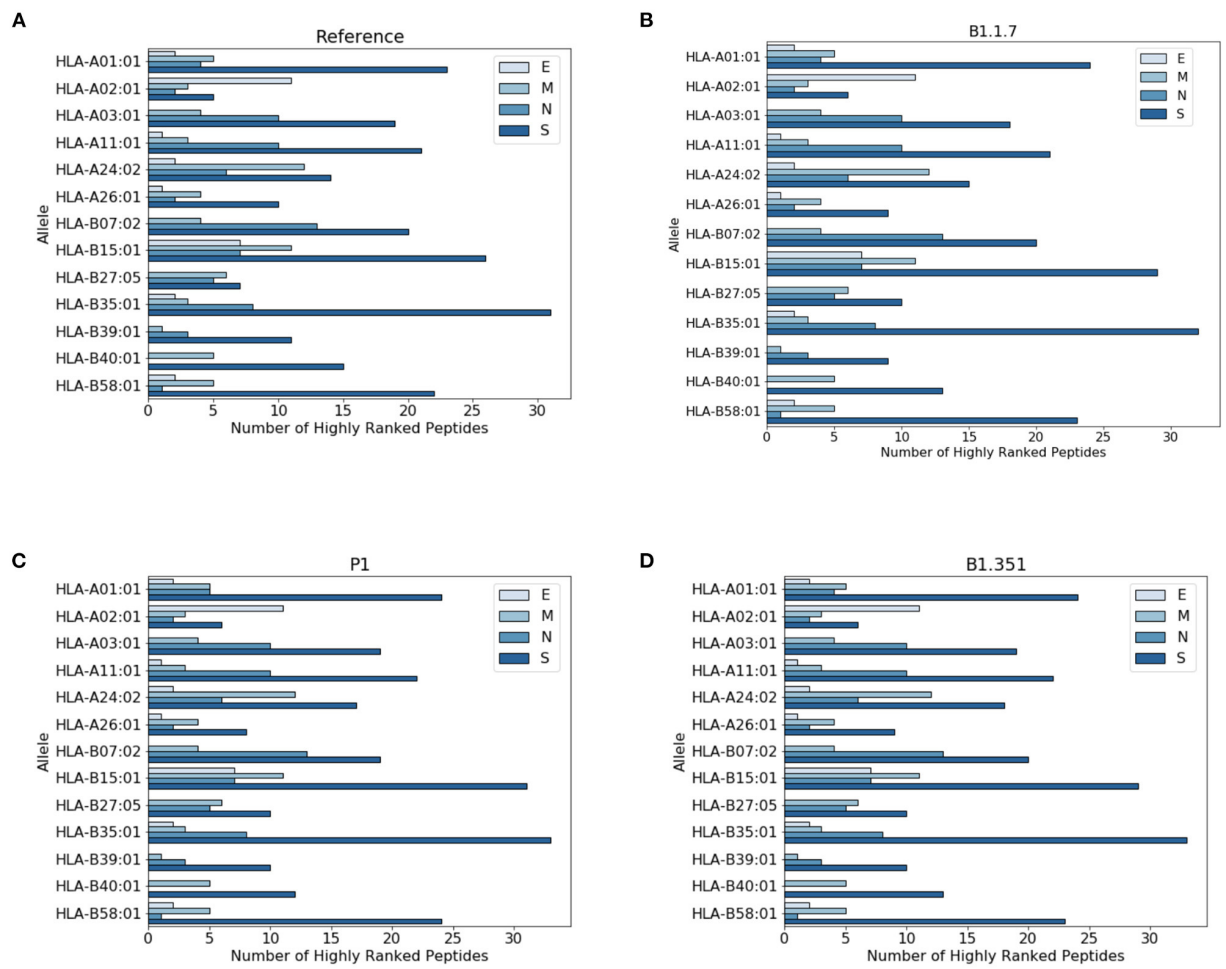
Distribution of highly ranked peptides across viral proteins. **(A)** Reference virus, **(B)** B1.1.7 **(C)** P1, and **(D)** B1.351.

In Figure 3 we report the protein localization of highly ranked peptides. Notice that most highly ranked peptides are located in the spike protein for all virus variants. We have also checked the localization of the new epitopes, not present in the reference virus. We found that virtually all the new epitopes are located in the spike protein, with a single exception of the P1 variant where one peptide stems from the mutated envelope protein.

## Discussion

Coronaviruses represent a broad class of viruses infecting humans through the upper respiratory tract and causing diseases with varying severity from common cold to flu-like diseases. SARS-CoV-2 has rapidly spread worldwide and has lead to thousands of mutations in a relatively short time, despite its low mutation rate. While most of these mutations do not carry any practical effect on the infection capability of the virus, some mutation can acquire higher transmissibility, the ability to better evade the immune system and stronger drug resistance (27–29). Three of these mutated strains, known as variants of concern (B.1.1.7, B.1.351, and P.1), have emerged and spread worldwide. Understanding the impact of mutations on viral infectivity and antigenicity is thus becoming a very pressing question (30). A recent paper showed that these mutations have only a small effect on SARS-CoV-2-specific CD4+ and CD8+ T cell responses in patients infected with the three virus variants (31).

In a recent paper (20), we have investigated the possible role of HLA type I polymorphism in SARS-CoV-2 susceptibility and we identified a set of peptides that were able to bind with high affinity a specific set of HLA type I alleles. We then studied the distribution of the relevant HLA type I alleles across human populations (20). Our conclusion was that the immune response may depend on the specific HLA class I haplotype of the infected subject. Therefore it is important to study the immune response to SARS-CoV-2 variants in an HLA-type I-dependent fashion.

In the present paper, we perform a computational analysis of the immune response to SARS-CoV-2 variants as compared with the original reference virus. Our results show that the number of potential peptides presented by HLA to T-cells varies depending on the HLA type I allele. While for some HLA class I alleles there is no change in the variant peptides with respect to the peptides in the reference virus, for some other HLA class I alleles the variation can be relatively large reaching more than 20% of the total. Our strategy can help screen for vaccine candidates that are robust against mutation. To design an effective vaccine, it is necessary to select peptides that can be presented to T cells by a range of HLAs that are broadly distributed in human populations. With our strategy one could also assess *in silico* if the peptides are still able to bind to HLAs when mutated.

## Data Availability Statement

The original contributions presented in the study are included in the article/Supplementary Material, further inquiries can be directed to the corresponding author/s.

## Author Contributions

CL and SZ designed and performed research and wrote the paper.

## Conflict of Interest

The authors declare that the research was conducted in the absence of any commercial or financial relationships that could be construed as a potential conflict of interest.
